# Optimization of screening strategies for colorectal cancer based on fecal DNA and occult blood testing

**DOI:** 10.1093/eurpub/ckad032

**Published:** 2023-03-11

**Authors:** Tingting Yao, Qin Sun, Kangwei Xiong, Yuan Su, Qian Zhao, Chenhong Zhang, Lijiu Zhang, Xuejun Li, Haiming Fang

**Affiliations:** Department of Gastroenterology, The Second Hospital of Anhui Medical University, Hefei, Anhui Province, China; Center of Gut Microbiota, The Second Hospital of Anhui Medical University, Hefei, Anhui Province, China; Department of Gastroenterology, The Second Hospital of Anhui University of Chinese Medicine, Hefei, Anhui Province, China; Department of Gastroenterology, The Second Hospital of Anhui Medical University, Hefei, Anhui Province, China; Center of Gut Microbiota, The Second Hospital of Anhui Medical University, Hefei, Anhui Province, China; Department of Gastroenterology, The Second Hospital of Anhui Medical University, Hefei, Anhui Province, China; Center of Gut Microbiota, The Second Hospital of Anhui Medical University, Hefei, Anhui Province, China; Department of Gastroenterology, The Second Hospital of Anhui Medical University, Hefei, Anhui Province, China; Center of Gut Microbiota, The Second Hospital of Anhui Medical University, Hefei, Anhui Province, China; Department of Gastroenterology, The Second Hospital of Anhui Medical University, Hefei, Anhui Province, China; Center of Gut Microbiota, The Second Hospital of Anhui Medical University, Hefei, Anhui Province, China; Department of Gastroenterology, The Second Hospital of Anhui Medical University, Hefei, Anhui Province, China; Center of Gut Microbiota, The Second Hospital of Anhui Medical University, Hefei, Anhui Province, China; Department of Gastroenterology, The Second Hospital of Anhui University of Chinese Medicine, Hefei, Anhui Province, China; Department of Gastroenterology, The Second Hospital of Anhui Medical University, Hefei, Anhui Province, China; Center of Gut Microbiota, The Second Hospital of Anhui Medical University, Hefei, Anhui Province, China

## Abstract

**Background:**

Fecal DNA and occult blood testing have been gradually developed for colorectal cancer (CRC) screening. Comparison of different testing strategies for these methods in CRC screening is in urgent need. This study aims to examine the efficacy of different testing strategies including multi-target fecal DNA testing, qualitative and quantitative fecal immunoassay tests (FITs).

**Methods:**

Fecal samples were collected from patients diagnosed by colonoscopy. Tests using fecal DNA, quantitative FIT or qualitative FIT were performed on same fecal samples. Efficiency of different testing strategies within different populations was investigated.

**Results:**

For high-risk populations (CRC and advanced adenoma), the positive rate of the three methods alone was 74.3–80%; the positive predictive values (PPVs) ranged from 37.3% to 77.8%, and the negative predictive values (NPVs) ranged from 86.3% to 92.2%. For combined testing strategies, the positive rate was 71.4–88.6%, PPVs ranged from 38.3% to 86.2%, and NPVs ranged from 89.6% to 92.9%. Parallel fecal multi-target DNA test and quantitative FIT appears to be superior when using a combined testing strategy. For the normal population, no significant difference was identified in efficacy between these methods when used alone and in combination.

**Conclusions:**

Single testing strategy among the three methods is more suitable for the general population screening, and the combined testing strategy is more suitable for high-risk populations screening. The use of different combination strategies may have superiority in CRC high-risk population screening, but cannot conclude significant differences which may be attributed to the small sample size, large samples controlled trials are needed.

## Introduction

Colorectal cancer (CRC) is the third most common cancer and the second leading cause of cancer-related deaths in the world, which accounts for nearly 1 in 10 new cancer cases diagnosed each year (∼14 million per year).^1^^,^^2^ CRC morbidity and mortality have been on the rise in China.^3^^,^^4^ Most sporadic CRC cases are transformed through an ‘adenoma-cancer’ pathway, which takes an average of 10–20 years.^5^ The cure rate of CRC in the early stage can reach more than 90%, whereas the 5-year survival rate of patients with advanced CRC is lower than 30%.^6^ Improving the testing rate of early CRC and precancerous lesions is an effective measure to improve the prognosis of CRC. CRC screening and early intervention has become an international consensus. In many developed countries, CRC screening has been regarded as a basic public medical service and has achieved remarkable economic and social benefits.^7–9^

Colonoscopy is the gold standard for CRC and adenoma screening, but it is an invasive procedure that even carries the risk of perforation. Before colonoscopy, patients are required to fast and drink large amounts of electrolyte solution for bowel preparation, resulting in a poor examination experience for patients. In addition, colonoscopy is a costly procedure in some countries. Although endoscopic screening and polypectomy could reduce the incidence and mortality of CRC,^10^ there is a high rate of missed diagnosis of colon lesions due to factors such as the quality of bowel preparation, the experience of the endoscopist, and the location, size and shape of the polyp or adenoma.^11^^,^^12^ About 3–8% of patients were still diagnosed with CRC 3–5 years after colonoscopy.^13^^,^^14^ Therefore, relatively accurate, convenient and noninvasive screening methods and screening strategies are urgently needed. Fecal testing is a widely used noninvasive screening method that is more acceptable to patients. The fecal occult blood test (FOBT) is the most commonly used fecal test and has been reported to reduce CRC-related mortality and potential CRC incidence.^15^ The FOBT evolved from chemical tests to immunochemical tests, and then from qualitative tests to quantitative tests, of which quantitative tests have attracted greater attention.^15^ In 1992, Sidransky *et al*.^16^ detected RAS mutations in the fecal DNA of patients with CRC, suggesting that fecal DNA testing could be used for CRC screening. Now, emerging evidences showed that fecal DNA testing mainly include: (i) gene methylation: TFPI2, BMP3, RARB2, NDRG4, APC, P16, GSTPL, HLTF, (ii) gene mutations: P53, K-RAS, MSI, APC. In the early stage, single gene mutation was detected, but the sensitivity was low. In recent years, studies have confirmed that multi-gene combined tests can improve the sensitivity of CRC screening.^17–19^ However, few studies have explored the values of different testing strategies for fecal DNA and fecal occult blood in CRC screening. This is a prospective study that evaluated the diagnostic efficacies of fecal multi-target DNA, fecal immunoassay test (FIT) qualitative and FIT quantitative testing in CRC screening, and compared the diagnostic efficacies of different combination of the three methods to find more sensitive and specific CRC screening strategies.

## Methods

### Subjects

Subjects admitted to the Department of Gastroenterology, the Second Hospital of Anhui Medical University and the Department of Gastroenterology, the Second Hospital of Anhui University of Chinese Medicine from April 2018 to October 2018 were prospective enrolled. Stool samples were collected from subjects for testing. All of the subjects eventually underwent colonoscopy. This study was approved by the ethics committee of the Second Hospital of Anhui Medical University and the Second Hospital of Anhui University of Traditional Chinese Medicine. All subjects signed written informed consent.

The inclusion criteria were as follows: the subjects ranged in age from 40 to 85, regardless of gender, and had no psychological, familial, social or geographic limitations that affected the study and follow-up compliance, volunteer to participate in the study and sign a written informed consent. The exclusion criteria were as follows: a history of CRC-related surgery, contraindications to colonoscopy, colonoscopy intolerance, reluctance to provide stool samples, participants who had received any drug trials within three months before the study, and a history of alcohol, drug or substance abuse. Participants who met the inclusion criteria but were subjectively unwilling to undergo colonoscopy were also excluded. According to the guidelines for follow-up after colorectal polypectomy, one of the following adenomas is considered to be an advanced adenoma and is defined as a high-risk adenoma: (i) adenoma ≥10 mm in diameter; (ii) villous adenoma or mixed adenoma with villous structure greater than 25%; and (iii) adenoma with high-grade intraepithelial neoplasia.^20^

### Research route diagram

Fecal samples were collected from patients diagnosed by colonoscopy. Tests using multi-target fecal DNA (New Horizon Health Co., Ltd, Hangzhou, China), FIT quantitative (Wodehealth Co., Ltd, Shenzhen China) and FIT qualitative (New Horizon Health Co., Ltd, Hangzhou China) were performed on same fecal samples. Efficiency of different testing strategies within different populations was investigated.

### Protocol for multi-target fecal DNA, FIT quantitative and qualitative testing

FIT tests include qualitative test and quantitative test. The principle is to use anti-human hemoglobin monoclonal antibody or polyclonal antibody to directly detect human hemoglobin in feces. The FIT qualitative test can directly show whether the hemoglobin detected in a fecal sample is above a specific threshold value, which is defined as positive or negative respectively. The FIT quantitative test is a direct measurement of fecal hemoglobin content: ≥100.0 ng/ml is defined as positive, <100.0 ng/ml is defined as negative. The fecal multi-target DNA test involves testing KRAS mutations, BMP3 and NDRG4 methylation, and hemoglobin levels in a fecal sample, and using logistic regression algorithms to generate a specific threshold value. According to the kit instructions, the threshold value is 165, <165 is defined as negative and ≥165 is positive. There was no quantitative linear relationship between the value and cancer progression.

### Colonoscopy

The patient fasted on the morning of the colonoscopy, and took 2000 ml of polyethylene glycol electrolyte solution for bowel preparation 4–6 h before the examination. A routine colonoscopy (PCF-H260, Olympus, Japan) was then performed, and a biopsy was performed simultaneously when CRC, adenoma or polyps were detected by the colonoscopy.

### Statistical analyses

SPSS 22.0 statistical software was employed for data analysis. Continuous variables were expressed as mean *±* standard deviation ($\overset{-}{x}$ ± *s*), and Student’s *t-*test or one-way ANOVA was used for comparison between groups. Categorical variables were quantified (percentage) and comparisons between groups were conducted using the χ^2^ test or Fisher’s exact test. Receiver Operating Characteristic (ROC) curve was used to evaluate the diagnostic value of fecal multi-target DNA testing, quantitative or qualitative FIT tesing, and different combinations of the three methods within different populations including CRC and advanced adenoma. *P *<* *0.05 was considered statistically significant.

## Results

### Demographic and clinical characteristics of the subjects

A total of 126 subjects were included in this study, including 78 males and 48 females, Thirteen patients aged 40–50, 49 patients aged 51–60, and 64 patients aged ≥61. Colonoscopies identified 20 CRCs, 24 adenomas, 35 non-neoplastic polyps and 47 normal subjects. The clinical features of the 20 cases of CRC were as follows: all patients were over 40 years old, with a male-female ratio of 15:5. Sixteen cases were located in the rectum, three in the sigmoid colon, and one in the transverse colon. Duck A, B, C and D stages were 3, 4, 7 and 6 cases, respectively. Ninety-five percent of the cases were adenocarcinoma (19/20) and one case was squamous cell carcinoma. The male-female ratio of the 24 adenomas was 18:6, including 15 advanced adenomas and 9 non-advanced adenomas. Of the 15 advanced adenomas, 11 were located in the rectum, 3 in the sigmoid colon and 1 in the ascending colon. The male–female ratio of the 35 patients with non-neoplastic polyps was 21:14. Thirty out of 35 patients (85.7%) aged between 50 and 69 years old. The male–female ratio of the 47 subjects with normal colonoscopies was 25:22, and 42/47 (93.3%) aged 50–59. Surveys of the subjects’ eating habits found that more than 90% of high-risk people, including those with CRC and advanced adenoma, preferred red meat. Approximately 45% of the low-risk group (including non-advanced adenomas and inflammatory and hyperplastic polyps) had a preference for red meat regularly, and about 55% had a preference for white meat.

### Characteristics of the three testing methods for colorectal cancer and different types of colorectal polyps

The positive rate of FIT qualitative was 90% (18/20) in CRC, 66.7% (10/15) in progressive adenoma, 44.4% (4/9) in non-progressive adenoma, 71.4% (25/35) in the non-neoplastic polyp and 38.3% (18/47) in the normal population, which showed significant differences (*P *<* *0.05). The positive rate of FIT quantitation in CRC was 100% (20/20), progressive adenoma was 40% (6/15), non-progressive adenoma was zero (0/9), hyperplastic and/or inflammatory polyps was 14.3% (5/35) and the normal population was 8.5% (4/47), with statistically significant differences (*P *<* *0.05). For the normal group, the false positive rate of FIT qualitative was significantly higher than that of FIT quantification (38.3% vs. 8.5%). The positive rate of multi-target fecal DNA testing in CRC was 100% (20/20), progressive adenoma was 53.3% (8/15), non-progressive adenoma was zero (0/9), proliferative and/or inflammatory polyps was 5.7% (2/35) and normal population was 12.8% (6/47), with statistically significant differences among the five groups (*P *<* *0.05), as shown in table 1.

**Table 1:** Comparison of three methods for testing of CRC and different types of polyps by single and combined testing

	FIT qualitative		FIT quantitative		Multi-target fecal DNA		FIT qualitative and multi-target fecal DNA		FIT qualitative or multi-target fecal DNA		FIT quantitativ and multi-target fecal DNA		FIT quantitative or multi-target fecal DNA	
	−	+	−	+	−	+	−	+	−	+	−	+	−	+
Normal colonoscopy	29	18 (38.3%)	43	4 (8.5%)	41	6 (12.8%)	44	3 (6.4%)	26	21 (44.7%)	45	2 (4.3%)	39	8 (17%)
Non-neoplastic polyps	10	25 (71.4%)	30	5 (14.3%)	33	2 (5.7%)	33	2 (5.7%)	10	25 (71.4%)	33	2 (5.7%)	30	5 (14.3%)
Non-progressive adenoma	5	4 (44.4%)	9	0 (0%)	9	0 (0%)	9	0 (0%)	5	4 (44.4%)	9	0 (0%)	9	0 (0%)
Progressive adenoma	5	10 (66.7%)	9	6 (40%)	7	8 (53.3%)	8	7 (46.7%)	4	11 (73.3%)	10	5 (33.3%)	6	9 (60%)
Colorectal cancer	2	18 (90%)	0	20 (100%)	0	20 (100%)	2	18 (90%)	0	20 (100%)	0	20 (100%)	0	20 (100%)

To verify the screening efficacy of different testing strategies, we analyzed the single use or combination of these three tests; the latter including pairwise parallel testing and pairwise series testing. A parallel positive test was defined as one positive test in a pairwise test, and a series positive test was defined as two simultaneous positive tests in a pairwise test. The results of the combined testing strategies for CRC and different types of colorectal polyps were shown in table 1. We found that the FIT qualitative and fecal DNA tandem tests significantly reduced the false positive rate in the normal group compared with the FIT qualitative alone test (6.4% vs. 38.3%). Compared with the FIT quantitative alone test, the FIT quantitative and fecal DNA tandem test also reduced the false positive rate in the normal group (4.3% vs. 8.5%). For CRC, the positive rates of the three methods used alone or in combination ranged from 90% to 100%.

### Comparison of the efficacy of the three methods used alone or in combination in high-risk and low-risk populations

The high-risk populations were defined as CRC and advanced adenomas, whereas the low-risk populations were defined as non-adenomatous polyps. In the high-risk populations, the positive rate of the three methods used alone was 74.3–80%. The PPVs ranged from 37.3% to 77.8%, and the negative predictive values (NPVs) ranged from 86.3% to 92.2%. In terms of single-use testing strategies, the fecal DNA multi-target and FIT quantitative testing showed higher screening efficiency, among while the fecal DNA multi-target testing performed the best (as shown in Supplementary tables S1 and S2).

**Figure 1:**
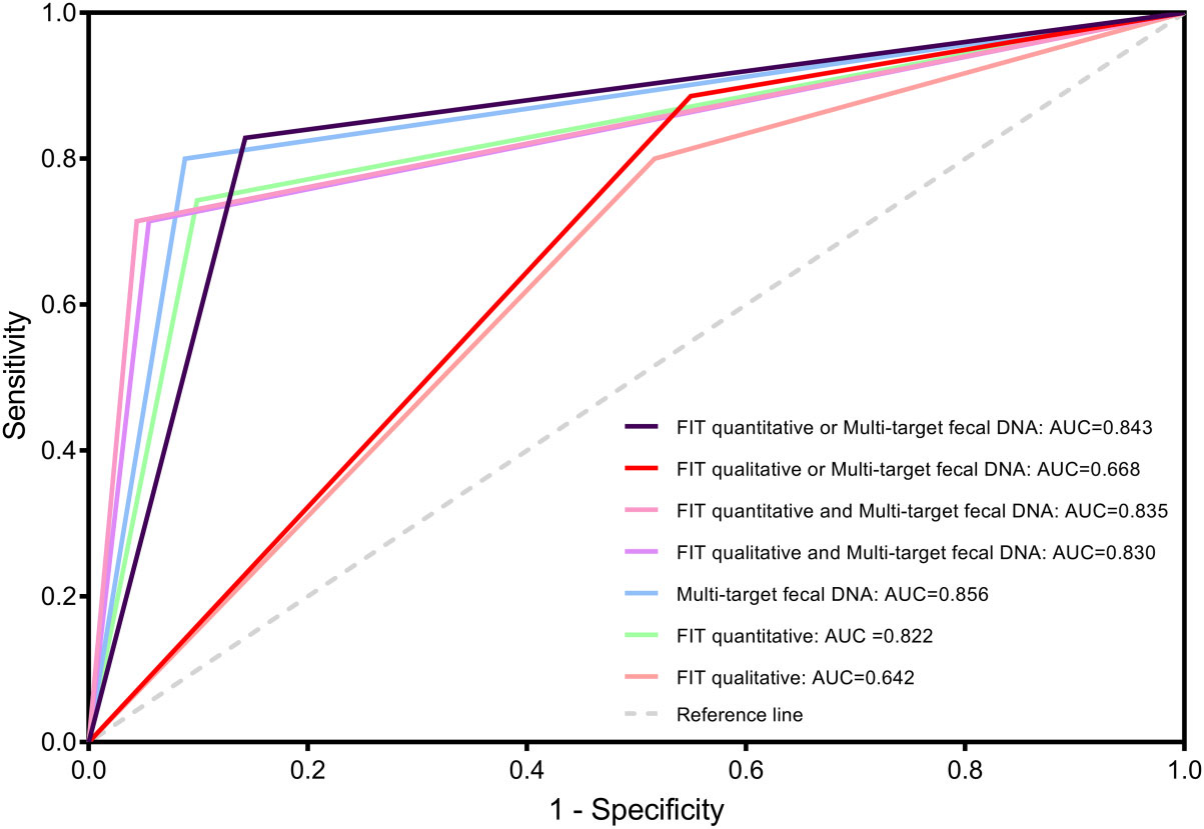
The ROC curves of the high-risk and low-risk populations were detected by the three methods alone and in combination

In terms of the combined testing strategy, the screening efficiency of fecal multi-target DNA and FIT qualitative parallel testing was the lowest. The screening efficiency of the parallel testing of fecal multi-target DNA and FIT quantitative were similar to that of the tandem test of fecal multi-target DNA and FIT qualitative or quantitative testing, among which the screening efficiency of parallel testing of fecal multi-target DNA and FIT quantitative testing presented slight superiority, as shown in Supplementary tables S1 and S2, and figure 1.

### Comparison of the efficacy of the three methods used alone or in combination in normal population and colorectal lesions populations

**Figure 2:**
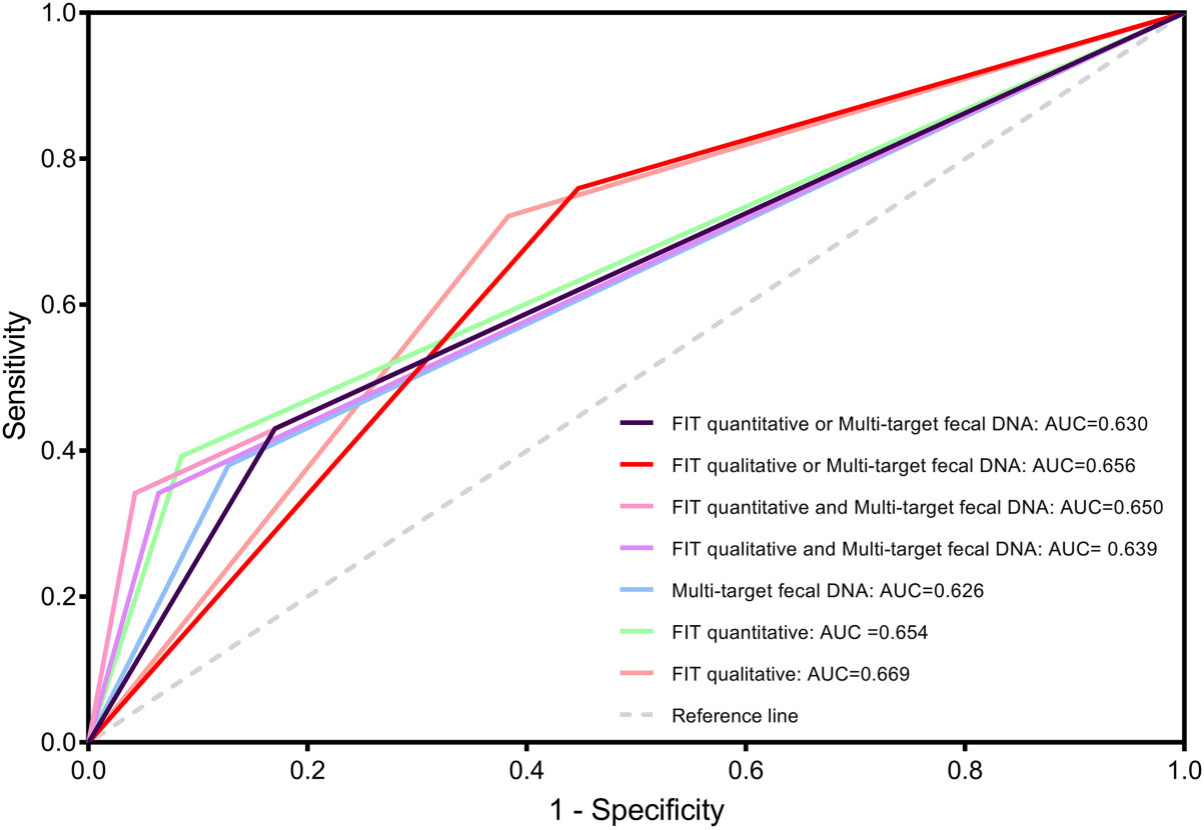
The ROC curves of the normal group and colorectal lesion group were detected by the three methods alone and in combination

The colorectal lesions populations included CRC, advanced adenomas and non-adenomatous polyps. The normal population means that the colonoscopy was normal. Similarly, to verify the efficacy of different testing strategies in colorectal lesions screening, we analyzed the single or combined use of these three tests, the latter including pairwise parallel testing and pairwise series testing. A parallel positive test was defined as one positive test in a pairwise test, and a series positive test was defined as two simultaneous positive tests in a pairwise test. We found that there were no significant differences among the screening efficacies when the three methods were used alone and in combination, suggesting that the single testing among the three methods is suitable for the general population screening, and the combined testing strategy is more suitable for high-risk population screening, as shown in Supplementary tables S3 and S4, and figure 2.

## Discussion

Early diagnosis and treatment can improve the diagnosis rate and survival rate of CRC, improve the testing rate of adenoma, and effectively block the process of adenoma carcinogenesis. Fecal multi-target DNA testing is included in the CRC screening guidelines recommended by the US Preventive Services Task Force (USPSTF) as a new screening method for CRC.^21^ Chinese expert consensus on early screening strategies for CRC agrees that fecal multi-target DNA testing is also recommended as one of the early screening techniques for CRC, and the recommended screening cycle is once every 3 years or once every 1 year.^22^ The US Joint Guidelines for CRC and Adenomatous Polyp Screening proposed that CRC screening should not only detect early cancer but also pay attention to the screening of adenomatous polyps.^23^ The Cologuard test had a sensitivity and specificity of 92% and 84%, respectively, for CRC. The sensitivity to detect advanced precancerous lesions (advanced adenomas and stemless serrated polyps with a diameter of 1 cm or greater) was 42%. The specificity of ‘all non-progressive results’, including non-tumor results and negative colonoscopy results, was 87%.^24^ In population screening, fecal DNA combined with FOBT increased the sensitivity of progressive adenomas to 42–54%.^25–27^

In this study, we found that the NPV of the three testing methods was between 86.3% and 92.2% when used alone, suggesting that when the testing results were negative, the prediction value for non-high-risk lesions was greater, which had important guiding value for the screening of CRC risk factors. The sensitivity and specificity of fecal DNA multi-target testing, including KRAS mutation, BMP3, and NDRG4 methylation, for CRC and advanced adenoma was 80%, 91.2%, and the corresponding area under the curve was 0.856. In terms of single-use testing strategies, multi-target fecal DNA and FIT quantitative testing had higher screening efficiency, among which multi-target fecal DNA testing showed the best effect. Fecal DNA testing is a noninvasive filter test for selecting relevant colonoscopy candidates, increasing compliance in the population to participate in CRC screening. However, fecal DNA screening is costly, and some uncertainty as to whether further diagnostic tests are necessary when the result is positive but the follow-up colonoscopy is negative.^28^^,^^29^

FIT is a test that uses monoclonal or polyclonal antibodies which can specifically distinguish the human globin in feces, which is not impacted by an individual’s diet or medications and does not present an abnormal result in the presence of upper gastrointestinal bleeding.^28^ The FIT qualitative test is convenient and quick, provide results in 5–10 min and cost less than screening colonoscopy and multi-target fecal DNA testing. In addition, sampling is convenient, not limited by location, and can be used for general screening of the population.

First, a FIT test can be used to qualitatively screen people at high risk of CRC, and those who test positive can be further tested for fecal DNA. If the FIT-DNA test is also positive, a colonoscopy is strongly recommended. A more detailed colonoscopy of patients who tested positive for FIT-DNA found the testing rate of flat colonic tumors in the right side was about three times higher than that in normal subjects.^30^ If the FIT-DNA test is negative, these patients should be followed up regularly and a colonoscopy should be completed if necessary. In terms of the combined testing strategy, the screening efficiency of the fecal multi-target DNA and FIT qualitative parallel testing was the lowest. The parallel testing of fecal multi-target DNA and FIT quantitative test and the tandem screening efficiency of fecal multi-target DNA and FIT qualitative or quantitative testing were similar, among which the screening efficiency of parallel testing of fecal multi-target DNA and FIT quantitative testing was the best. To the best of our knowledge, this study is the first to use fecal DNA test in combination with the FIT test to form a multi-modal CRC screening strategy, which can improve the screening sensitivity of both CRC and progressive adenoma.

In addition, literature evidences propose an association between red meat consumption and CRC development, and red meat is positively correlated with CRC. Degradation products of red meat allow for the creation of a pro-inflammatory colonic microenvironment, and the gut microbiome plays a role in colorectal carcinogenesis.^31^^,^^32^ In the present study, we found that more than 90% of the high-risk population and 45% of the low-risk population habitually consumed red meat, further supporting the association between red meat and CRC. The stool bacterial load is higher in individuals with high-grade dysplasia and CRC, and the value of fecal bacterial markers in CRC screening is gaining attention.^28^ However, stool-based microbiome tests are emerging screening modalities that are not yet FDA-approved or recommended for average-risk screening.^28^

## Conclusion

The single testing strategy among the three methods is more suitable for the general screening of the population, and the combined testing strategy is more suitable for high-risk populations screening. Different combination strategies seem to present some superiority in CRC high-risk population screening, but there were no significant differences among the combinatorial methods, which may be attributed to the small sample size. This needs to be further verified by rigorous controlled trials with large samples.

### Limitations

This study is pilot study with a small sample size. Although it was found that fecal multi-target DNA combined with FIT quantitative tandem testing seemed to present a certain degree of superiority in CRC high-risk population screening, no significant difference among the three combination strategies was identified. Further clinical trials with large sample size are to be conducted.

## Supplementary Material

ckad032_Supplementary_Data

## Data Availability

The data underlying this article will be shared on reasonable request to the corresponding author.
