# Supplementary material for: Optimization of screening strategies for colorectal cancer based on fecal DNA and occult blood testing
Source: Eur J Public Health. 2023 Mar 11;33(2):336–41. doi: 10.1093/eurpub/ckad032 (PMC10066493; doi:10.1093/eurpub/ckad032)
Supplement: ckad032_Supplementary_Data [file ckad032_supplementary_data.docx]

**S-Table 1 Comparison of three methods for high risk group and low risk population by single and combined detection**

|  | FIT qualitative | | FIT quantitative | | Multi-target fecal DNA | | FIT qualitativ＆Multi-target fecal DNA | | FIT qualitative or Multi-target fecal DNA | | FIT quantitativ＆Multi-target fecal DNA | | FIT quantitative or Multi-target fecal DNA | |
| --- | --- | --- | --- | --- | --- | --- | --- | --- | --- | --- | --- | --- | --- | --- |
|  | - | + | - | + | - | + | - | + | - | + | - | + | - | + |
| Low risk | 44 | 47（51.6%） | 82 | 9（9.9%） | 83 | 8（8.8%） | 86 | 5（5.5%） | 41 | 50（54.9%） | 87 | 4（4.4%） | 78 | 13（14.3%） |
| High risk | 7 | 28（80%） | 9 | 26（74.3%） | 7 | 28（80%） | 10 | 25（71.4%） | 4 | 31（88.6%） | 10 | 25（71.4%） | 6 | 29（82.9%） |

Note: high risk group was defined as CRC and advanced adenomas, low risk group was defined as non-adenomatous polyps

**S-Table 2 Comparison of the efficacy of single and combined detection of three methods in high risk and low risk population**

|  | Sensitivity | Specificity | PPV | NPV | AUC |
| --- | --- | --- | --- | --- | --- |
| FIT qualitative | 80% | 48.4% | 37.3% | 86.3% | 0.642 |
| FIT quantitative | 74.3% | 90.1% | 74.3% | 90.1% | 0.822 |
| Multi-target fecal DNA | 80% | 91.2% | 77.8% | 92.2% | 0.856 |
| FIT qualitativ＆Multi-target fecal DNA | 71.4% | 94.5% | 83.3% | 89.6% | 0.830 |
| FIT qualitative or Multi-target fecal DNA | 88.6% | 44.1% | 38.3% | 91.1% | 0.668 |
| FIT quantitativ＆Multi-target fecal DNA | 71.4% | 95.6% | 86.2% | 89.7% | 0.835 |
| FIT quantitative or Multi-target fecal DNA | 82.9% | 85.7% | 69.0% | 92.9% | 0.843 |

Note: high risk group was defined as CRC and advanced adenomas, low risk group was defined as non-adenomatous polyps. PPV, positive predictive value,

NPV,negative predictive value, AUC, area under the curve

**S-Table 3 Comparison of three methods for normal group and colorectal lesion group by single and combined detection**

|  | FIT qualitative | | FIT quantitative | | Multi-target fecal DNA | | FIT qualitativ＆Multi-target fecal DNA | | FIT qualitative or Multi-target fecal DNA | | FIT quantitativ＆Multi-target fecal DNA | | FIT quantitative or Multi-target fecal DNA | |
| --- | --- | --- | --- | --- | --- | --- | --- | --- | --- | --- | --- | --- | --- | --- |
|  | - | + | - | + | - | + | - | + | - | + | - | + | - | + |
| Normal colonoscopy | 29 | 18（38.3%） | 43 | 4（8.5%） | 41 | 6（12.8%） | 44 | 3（6.4%） | 26 | 21（44.7%） | 45 | 2（4.3%） | 39 | 8（17%） |
| colorectal lesions | 22 | 57（72.2%） | 48 | 31（39.2%） | 49 | 30（38%） | 52 | 27（34.2%） | 19 | 60（75.9%） | 52 | 27（34.2%） | 45 | 34（43%） |

**S-Table 4 Comparison of the efficacy of single and combined detection of three methods in normal group and colorectal lesion group**

|  | Sensitivity | Specificity | PPV | NPV | AUC |
| --- | --- | --- | --- | --- | --- |
| FIT qualitative | 72.2% | 66.2% | 76% | 56.9% | 0.669 |
| FIT quantitative | 39.2% | 91.5% | 88.6% | 47.3% | 0.654 |
| Multi-target fecal DNA | 38% | 87.2% | 83.3% | 54.4% | 0.626 |
| FIT qualitativ＆Multi-target fecal DNA | 34.2% | 93.6% | 90% | 45.8% | 0.639 |
| FIT qualitative or Multi-target fecal DNA | 75.9% | 55.3% | 74.1% | 57.8% | 0.656 |
| FIT quantitativ＆Multi-target fecal DNA | 34.2% | 95.7% | 90% | 46.4% | 0.650 |
| FIT quantitative or Multi-target fecal DNA | 43% | 83% | 80.1% | 46.4% | 0.630 |

Note: PPV, positive predictive value, NPV,negative predictive value, AUC, area under the curve
